# Endoscopic Ultrasound: Reaching Where Others Can’t

**DOI:** 10.7759/cureus.1169

**Published:** 2017-04-15

**Authors:** Fady G. Haddad, Magda Daoud, Ying Liu, Sherif Andrawes

**Affiliations:** 1 Department of Internal Medicine, Staten Island University Hospital; 2 Pathology and Laboratory Medicine, Staten Island University Hospital; 3 Department of Gastroenterology, Staten Island University Hospital

**Keywords:** endoscopic ultrasound, pancreatic tail lesion

## Abstract

Endoscopic ultrasound (EUS) has been increasingly used for the diagnosis and staging of pancreatic cancer. It has recently become the modality of choice in assessing pancreatic lesions overcoming other traditional modalities. Typically lesions located at the tail of the pancreas are best accessed through the stomach. We present a patient with pancreatic tail mass occurring in the setting of a large hiatal hernia, intrathoracic stomach, and severe lumbar levoscoliosis. Due to altered anatomy and extensive vascular connections of the mass, any surgical or radiological intervention was considered high risk for the patient. EUS was the only modality capable of providing a pancreatic mass tissue sample in this patient with challenging thoraco-abdominal anatomy. Moreover, pancreatic tail lesions are traditionally best accessed through the gastric fundus; however, in view of the patient’s altered anatomy, EUS-fine needle aspiration (FNA) had to be performed through the duodenum. This case raises the importance of EUS when surgical and radiological interventions are restricted.

## Introduction

Endoscopic ultrasound (EUS) has been increasingly used for the diagnosis and staging of pancreatic cancer [1]. It has recently become the modality of choice in assessing pancreatic lesions overcoming other traditional modalities [1]. Lesions located at the tail of the pancreas are typically accessed through the stomach [2]. Conversely, we present a patient with pancreatic tail mass in the setting of a severely altered anatomy and extensive vascular connections where the lesion had to be accessed through the duodenum. Moreover, EUS was the only modality capable of providing a tissue sample in this patient in view of the limitations of radiological and surgical interventions.

## Case presentation

**Figure 1:**
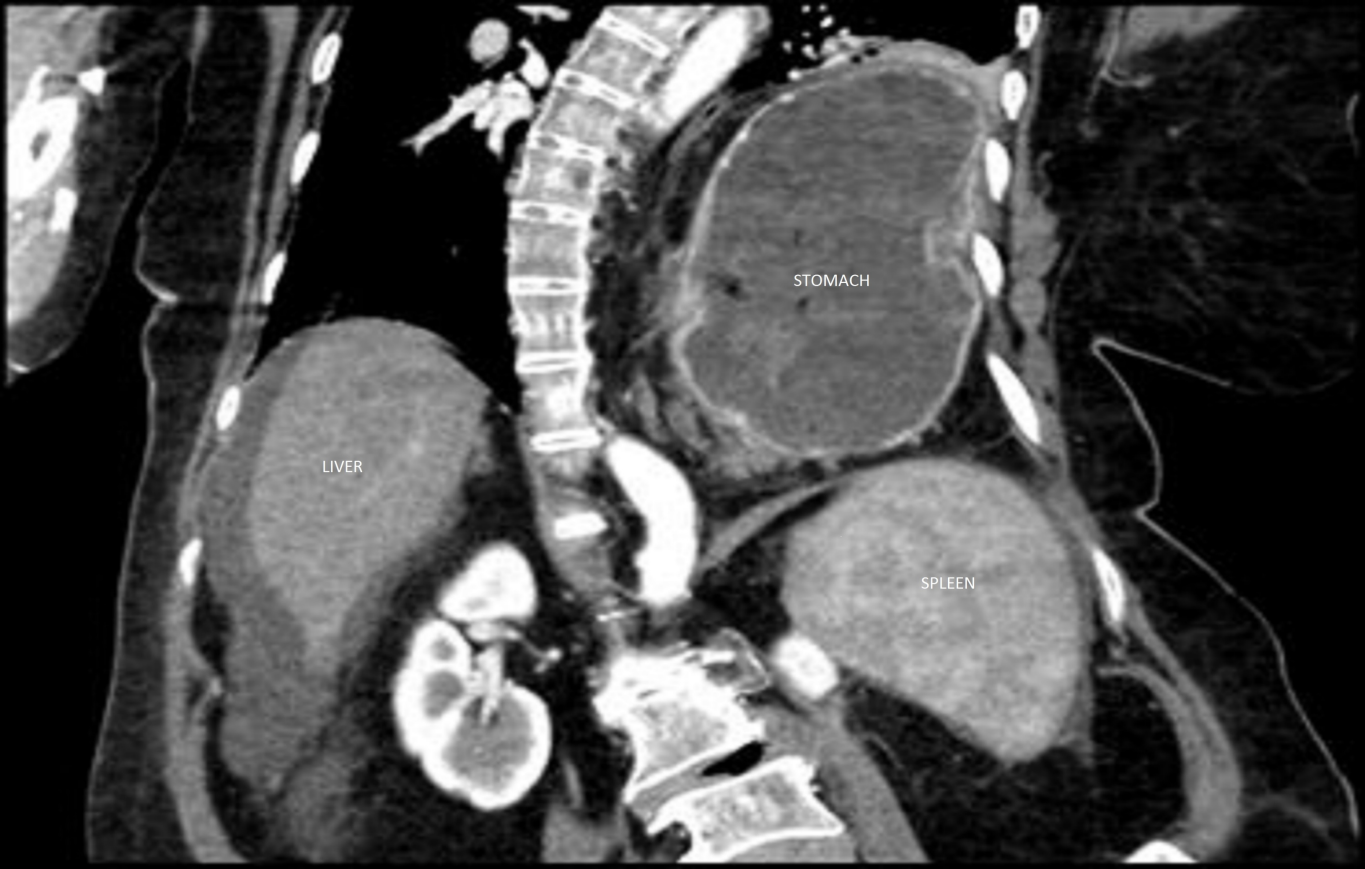
Contrast abdominal computed tomography (CT) scan. Coronal view showing an elevated left hemidiaphragm and a large hiatal hernia containing the stomach. The osseous structures demonstrate severe lumbar levoscoliosis and degenerative changes of the spine. Moderate ascites and splenomegaly were also noted.

A 61-year-old woman with a known large hiatal hernia with intrathoracic stomach presented with a few weeks' history of isolated epigastric pain. Past medical history was otherwise significant for severe lumbar levoscoliosis (Figure 1) and hepatitis C virus-related liver cirrhosis.

**Figure 2:**
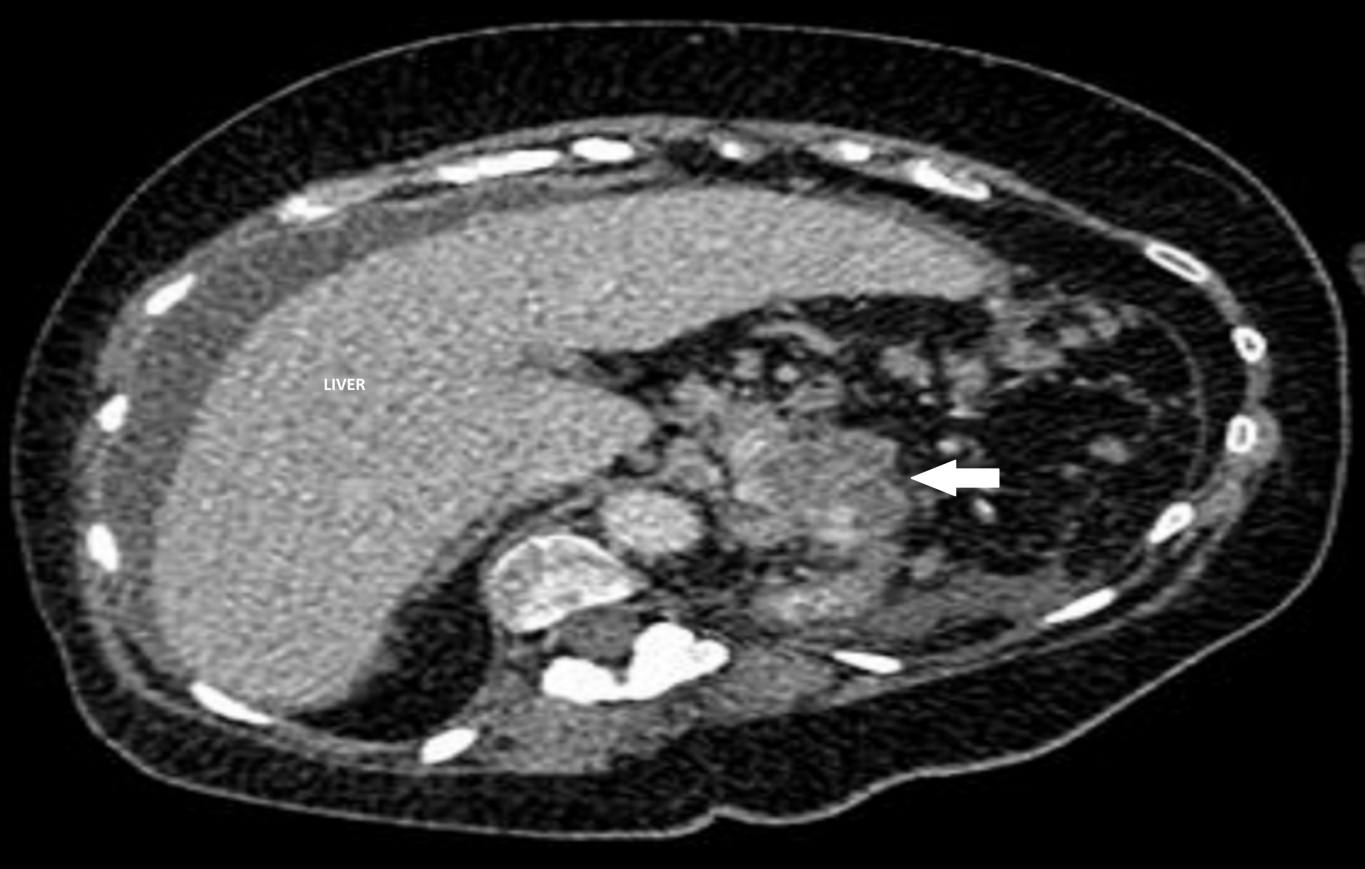
Contrast abdominal computed tomography (CT) scan. Axial view showing an irregularly marginated 3.0 x 3.0 cm heterogeneous hypodense mass (arrow) nearly completely replacing the distal pancreas, suspicious for pancreatic adenocarcinoma.

Abdominal computed tomography (CT) scan noted an irregular 3.0 × 3.0 cm heterogeneous mass (Figure 2) arising from the pancreatic tail, inseparable from the splenic artery along its entire course and contacting a short segment of the splenic vein at its inferior part.

**Figure 3:**
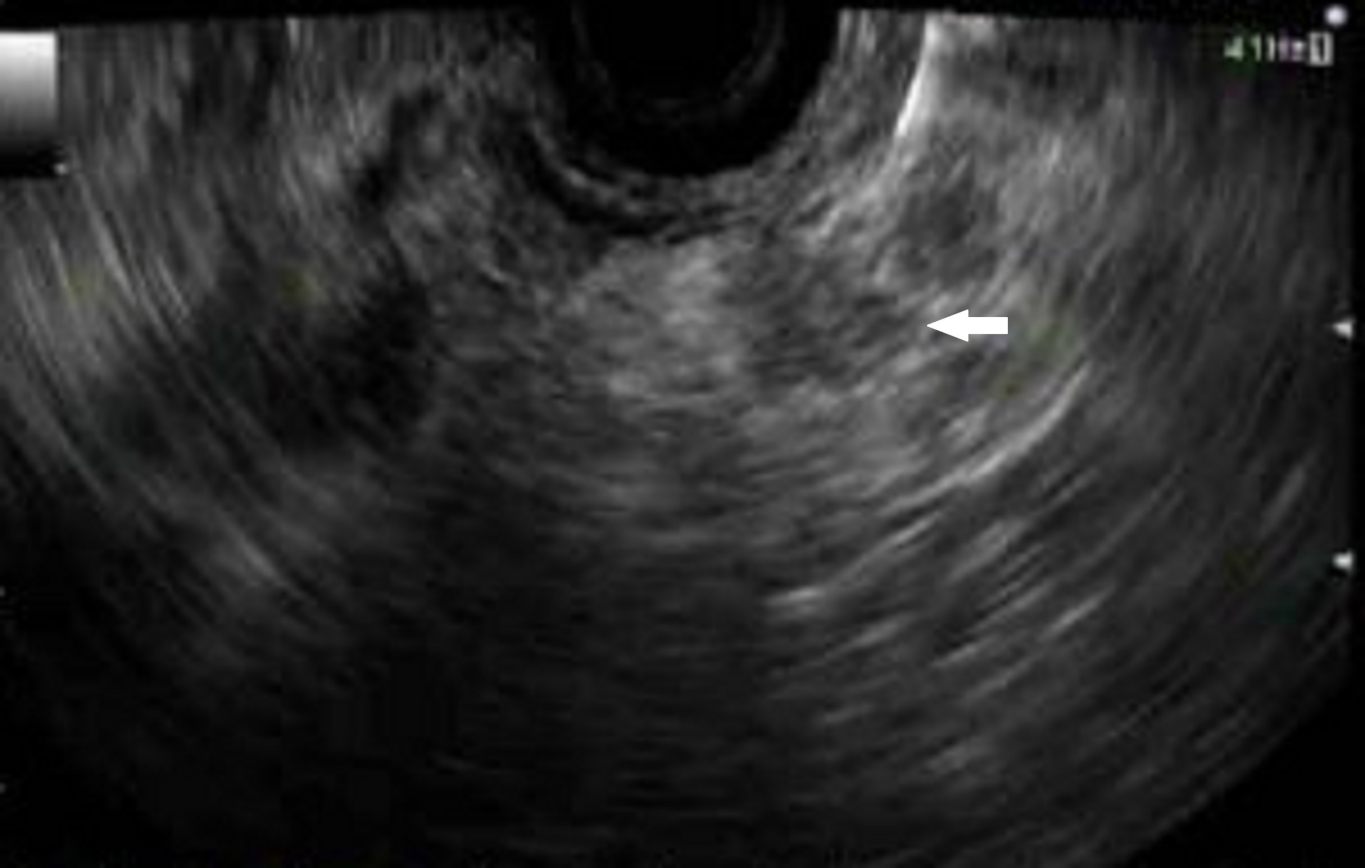
Endoscopic ultrasound image showing a 3.0 x 3.0 cm hypoechoic mass lesion in the pancreatic tail (arrow). The mass was invading into the splenic vasculature. There were multiple malignant-appearing lymph nodes in the peripancreatic area.

Due to abnormal anatomy and extensive mass vascularization, any surgical or interventional radiology procedure was considered high risk for the patient. EUS was requested to provide histopathological diagnosis. Upper endoscopy outlined the anatomy showing a large hiatal hernia with an intrathoracic stomach. A guidewire had to be inserted to facilitate the passage of the EUS scope into the duodenum. The linear echoendoscope was advanced down to the duodenal bulb. A 3.0 × 3.0 cm pancreatic tail mass was identified (Figure 3) with the invasion of the splenic vasculature and notable malignant appearing lymph nodes.

**Figure 4:**
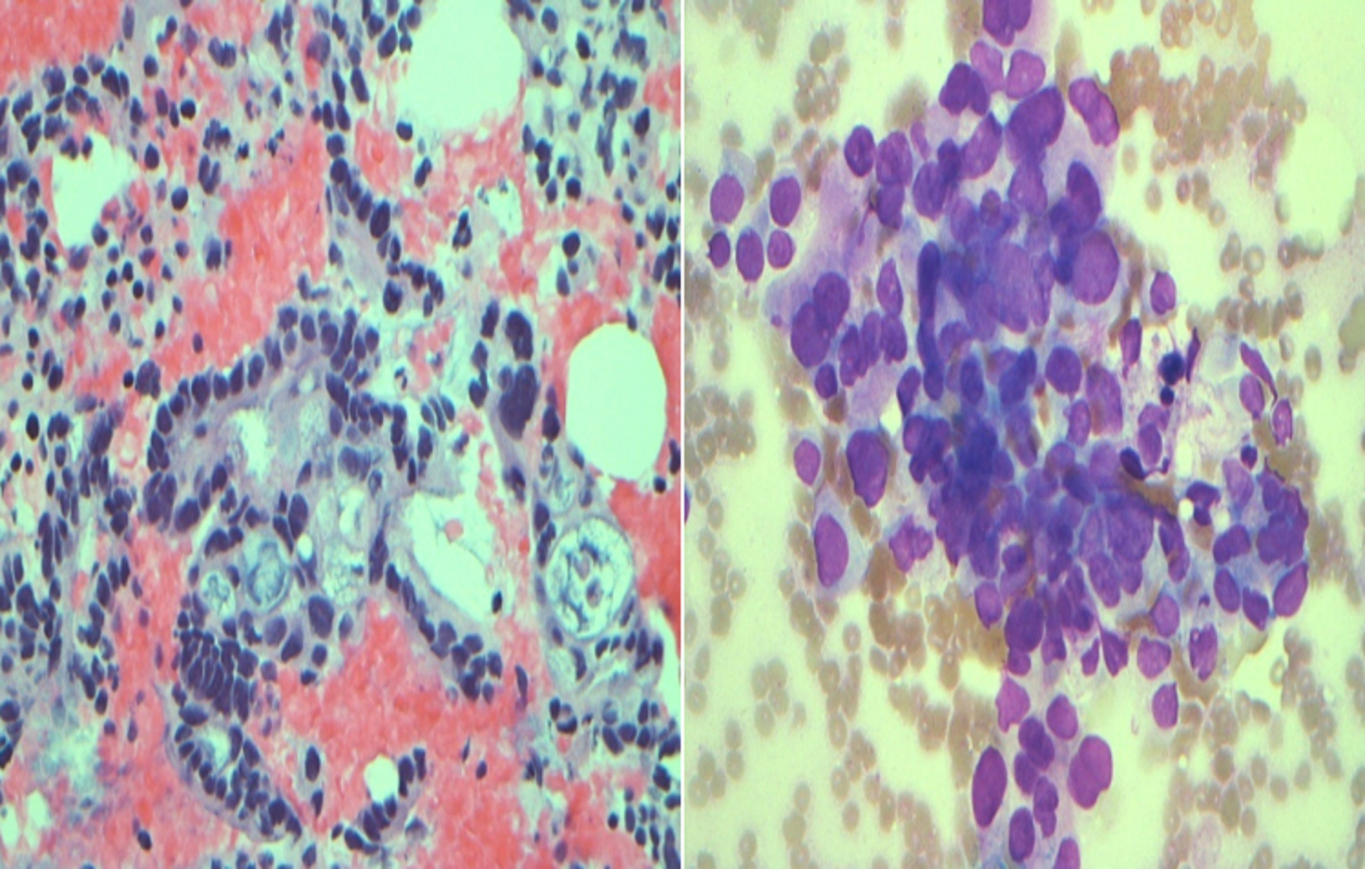
(Left): 40X Hematoxylin and Eosin stained cell block showing clusters of malignant cells with cytological atypia including variation of nuclear size and shape and irregular nuclear contours consistent with adenocarcinoma. (Right): 40X Diff-quick stained smear showing clusters of malignant cells with cytological atypia including crowding, nuclear overlapping, variation in size and shape of the nuclei and irregular nuclear contours consistent with adenocarcinoma.

Fine needle aspiration was successfully performed using a 25 gauge needle through two separate passes through the duodenal wall. Cytology results confirmed the diagnosis of pancreatic adenocarcinoma (Figure 4 left, right).

## Discussion

Tissue sampling is a major step in the diagnosis and management of pancreatic lesions [3]. Pancreatic masses could only be accessed through conventional techniques in the past, namely percutaneous and CT-guided biopsies in addition to endoscopic retrograde cholangiopancreatography (ERCP) with brush cytology [3]. However, EUS has recently overcome the other traditional modalities in assessing pancreatic lesions in view of its higher diagnostic yield, lower cost, technical ease, and improved safety profile [3-5]. In a recent meta-analysis, EUS-fine needle aspiration (FNA) was found to have a sensitivity of 85% and a specificity of 98% in diagnosing pancreatic cancer [3]. If the initial procedure is nondiagnostic, it is recommended to repeat EUS-FNA given the improved diagnostic yield with repeated interventions [6]. Several factors can affect the accuracy of EUS-FNA including adequacy of tissue sample, location of the mass, endoscopist’s expertise, in addition to on-site cytopathology availability and presence of chronic pancreatitis [7-9]. Limitations of EUS-FNA include the inability to obtain a histologic architecture of the acquired tissue and an indeterminate number of passes needed to attain an adequate sample in the absence of an on-site cytopathologist [3]. Recent trials have suggested that EUS-guided fine needle biopsy (FNB) might be able to overcome these limitations [3]. EUS-FNA has a complication rate of one to two percent [3]. Complications occur more often with pancreatic cystic rather than solid lesions and mainly include pancreatitis, bleeding, perforation, infection, and tumor seeding [6]. Traditionally pancreatic tail lesions are best accessed through the gastric fundus as this avoids scope angulation allowing an easy passage of the needle through the biopsy channel [6]. However, in view of our patient’s altered anatomy and intrathoracic location of the stomach, EUS-FNA had to be performed through the duodenum [2].

## Conclusions

We present a case where EUS was the only modality capable of providing a pancreatic mass tissue sample in a patient with a challenging thoracoabdominal anatomy. This case raises the importance of EUS when surgical and radiological interventions are restricted.
